# Supplementary material for: Real-Time Humidity Measurement during Sports Activity using Optical Fibre Sensing
Source: Sensors (Basel). 2020 Mar 30;20(7):1904. doi: 10.3390/s20071904 (PMC7181039; doi:10.3390/s20071904)
Supplement: Supplementary file 1 [file sensors-20-01904-s001.pdf]

Supplementary

Real-time Humidity Measurement in Sports Activity using Optical Fibre Sensing

C. He[1], S. Korposh[1], F.U. Hernandez[2], L. Liu[1], R. Correia[1], B.R. Hayes-Gill[1] and S.P. Morgan[1,2]*

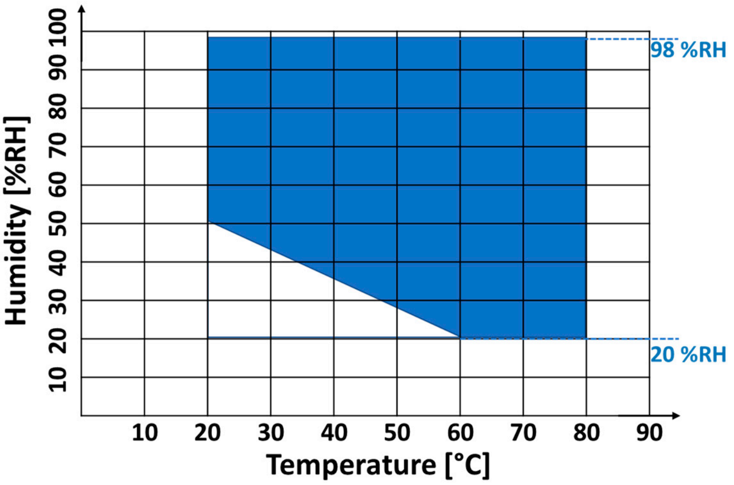

Figure S1. Temperature humidity control range of the climatic chamber.

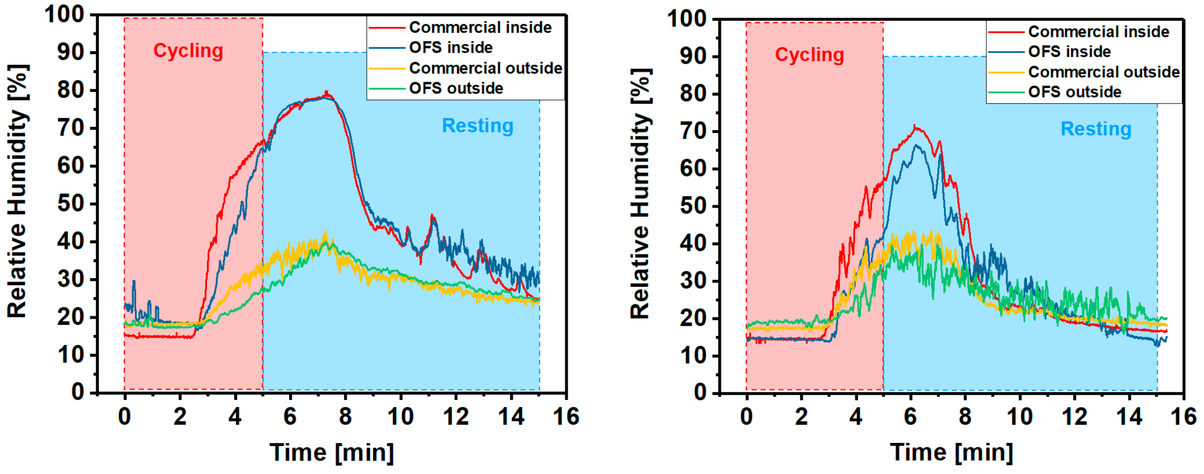

(a)

(b)

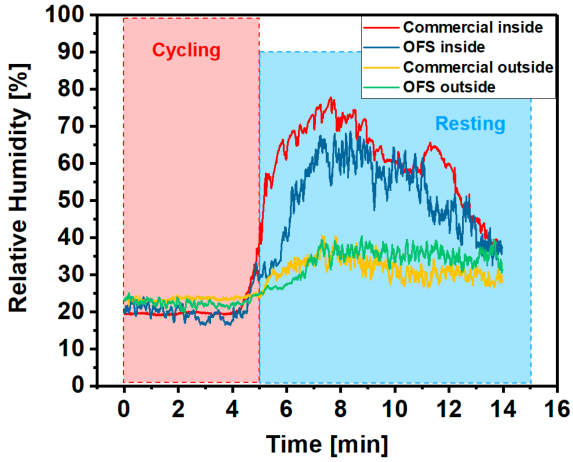
Relative Humidity [%]
Time [min]
Cycling
Resting
Commercial inside
OFS inside
Commercial outside
OFS outside

(c)

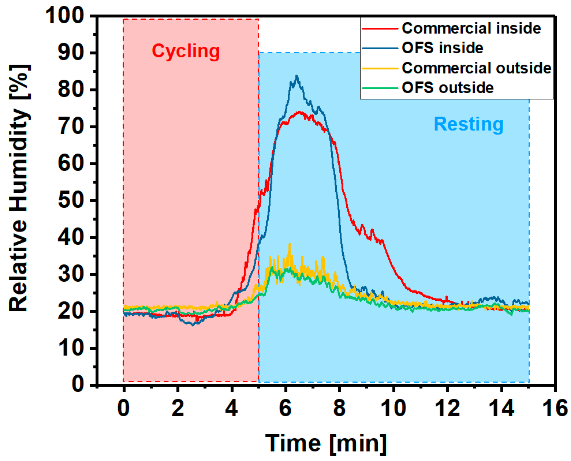
Relative Humidity [%]
Time [min]
Cycling
Resting
Commercial inside
OFS inside
Commercial outside
OFS outside

(d)

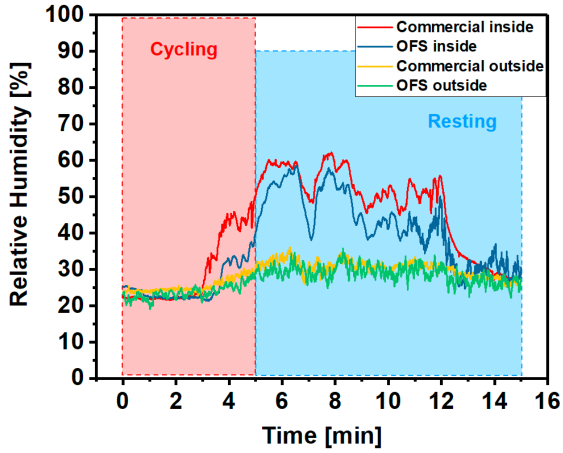
Relative Humidity [%]
Time [min]
Cycling
Resting
Commercial inside
OFS inside
Commercial outside
OFS outside

(e)

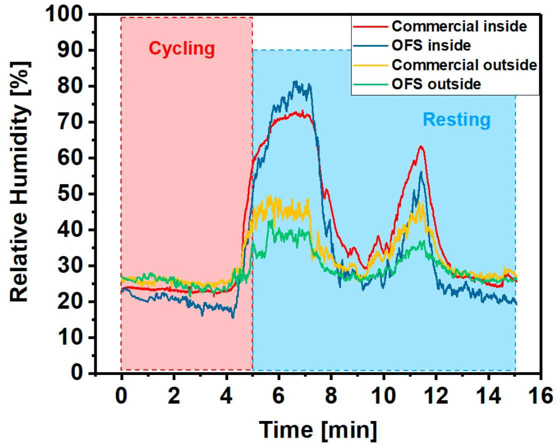
Relative Humidity [%]
Time [min]
Cycling
Resting
Commercial inside
OFS inside
Commercial outside
OFS outside

(f)

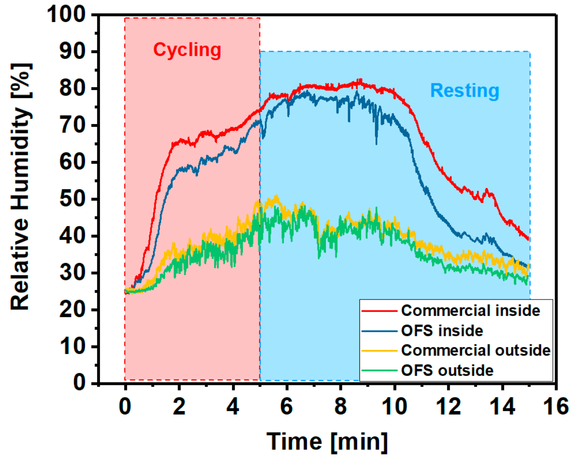
Relative Humidity [%]
Time [min]
Cycling
Resting
Commercial inside
OFS inside
Commercial outside
OFS outside

(g)

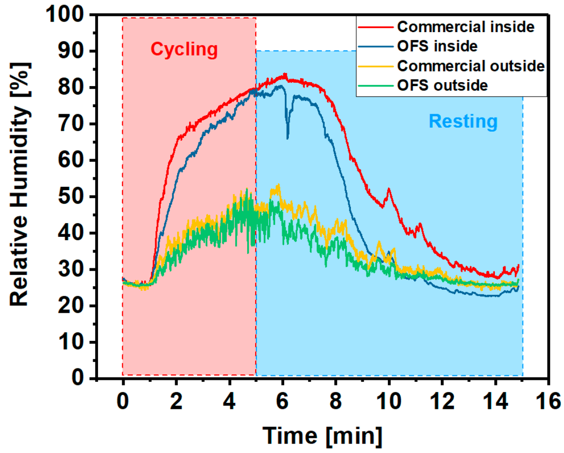
Relative Humidity [%]
Time [min]
Cycling
Resting
Commercial inside
OFS inside
Commercial outside
OFS outside

(h)

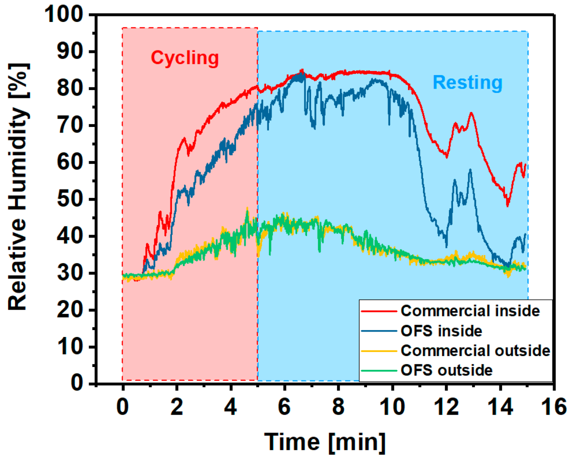
Relative Humidity [%]
Time [min]
Cycling
Resting
Commercial inside
OFS inside
Commercial outside
OFS outside

(i)

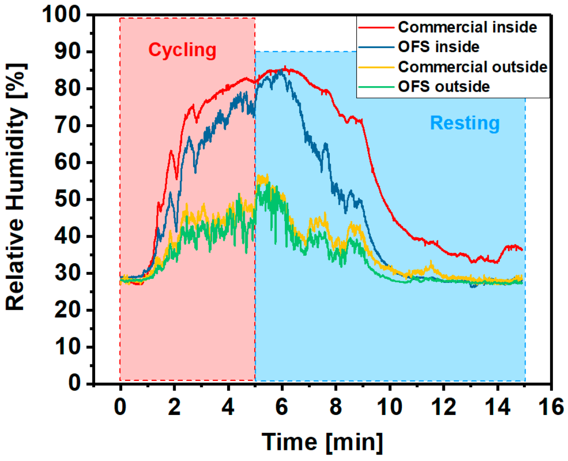
Relative Humidity [%]
Time [min]
Cycling
Resting
Commercial inside
OFS inside
Commercial outside
OFS outside

(j)

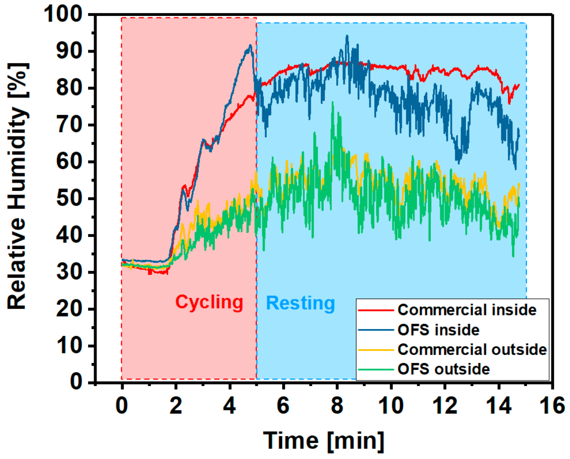
Relative Humidity [%]
Time [min]
Cycling
Resting
Commercial inside
OFS inside
Commercial outside
OFS outside

(k)

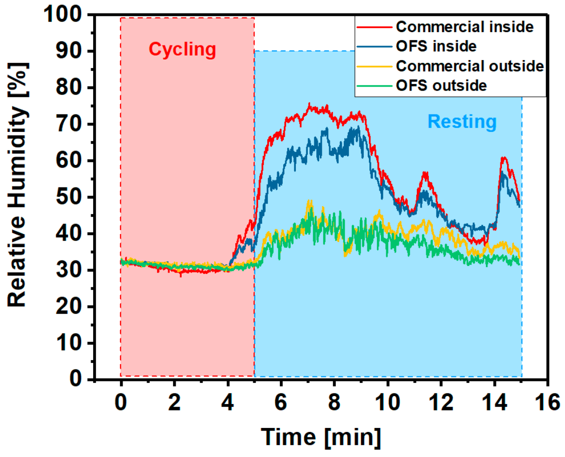
Relative Humidity [%]
Time [min]
Cycling
Resting
Commercial inside
OFS inside
Commercial outside
OFS outside

(l)

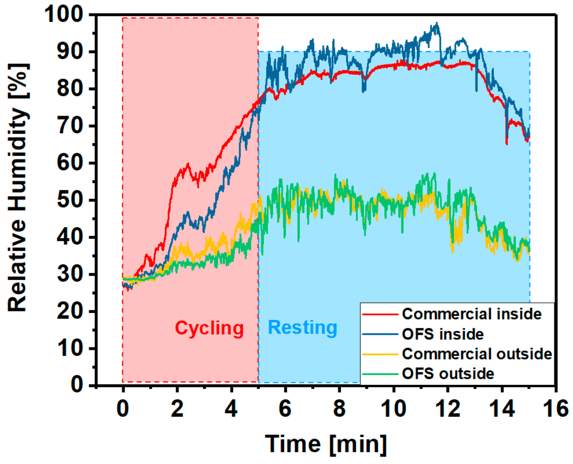
Relative Humidity [%]
Time [min]
Cycling
Resting
Commercial inside
OFS inside
Commercial outside
OFS outside

(m)

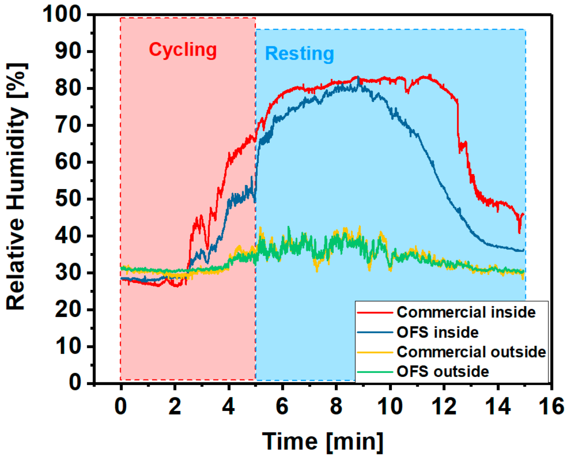
Relative Humidity [%]
Time [min]
Cycling
Resting
Commercial inside
OFS inside
Commercial outside
OFS outside

(n)

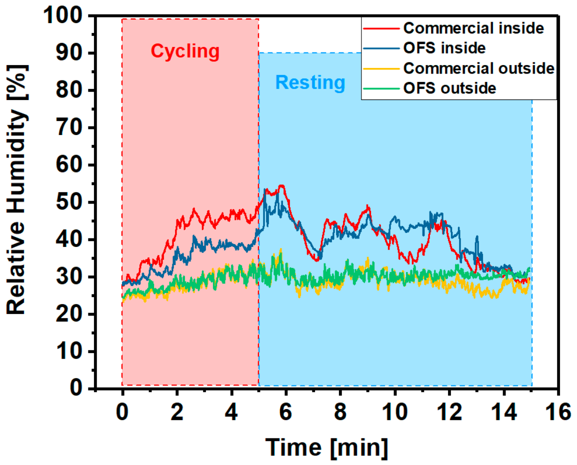

(o)

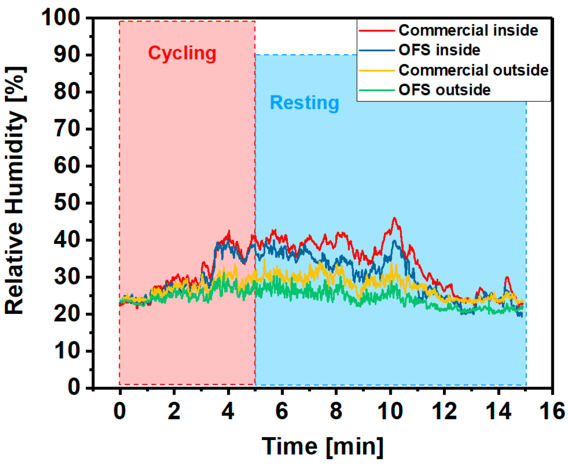

(p)

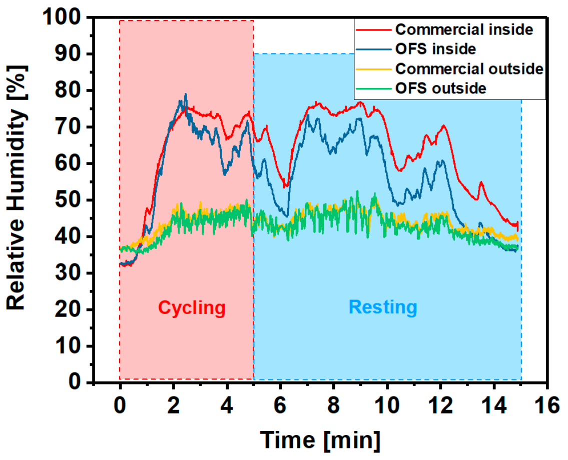

(q)

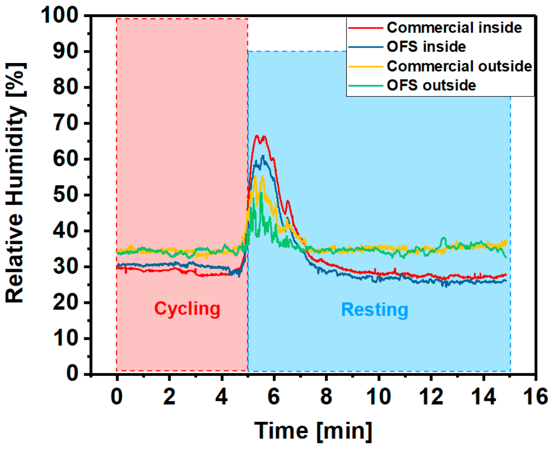

(r)

Figure S2. The RH readings from the sensing probe (the RH reading from the inner sensing probe is indicated by the blue line, and the RH reading from the outer sensing probe is indicated by the green line) and the commercial sensor (the RH reading from the inner commercial sensor is indicated by the red line, and the RH reading from the outer commercial sensor is indicated by the yellow line) measured during the cycling exercise for volunteer 2 with a 100% cotton T-shirt (**a**) and a 100% polyester T-shirt (**b**), for volunteer 3 with a 100% cotton T-shirt (**c**) and a 100% polyester T-shirt (**d**), for volunteer 4 with a 100% cotton T-shirt (**e**) and a 100% polyester T-shirt (**f**), for volunteer 5 with a 100% cotton T-shirt (**g**) and a 100% polyester T-shirt (**h**), for volunteer 6 with a 100% cotton T-shirt (**i**) and a 100% polyester T-shirt (**j**), for volunteer 7 with a 100% cotton T-shirt (**k**) and a 100% polyester T-shirt (**l**), for volunteer 8 with a 100% cotton T-shirt (**m**) and a 100% polyester T-shirt (**n**), for volunteer 9 with a 100% cotton T-shirt (**o**) and a 100% polyester T-shirt (**p**), for volunteer 10 with a 100% cotton T-shirt (**q**) and a 100% polyester T-shirt (**r**).

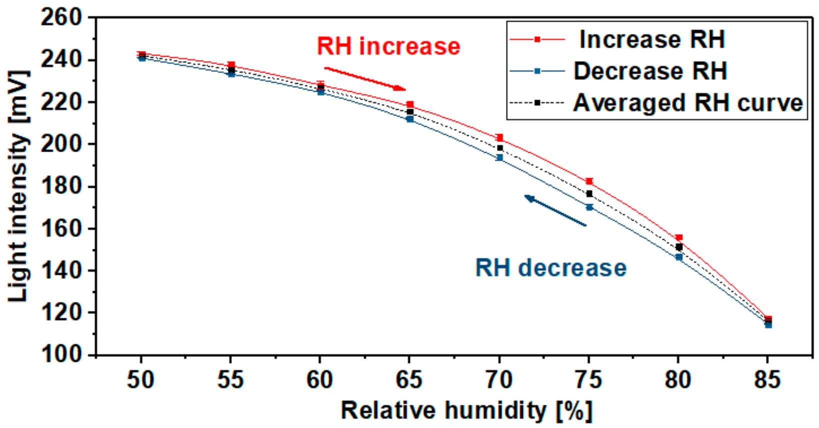

Figure S3. OFHS responses with the increase and decrease of RH. Error bars are smaller than the markers.

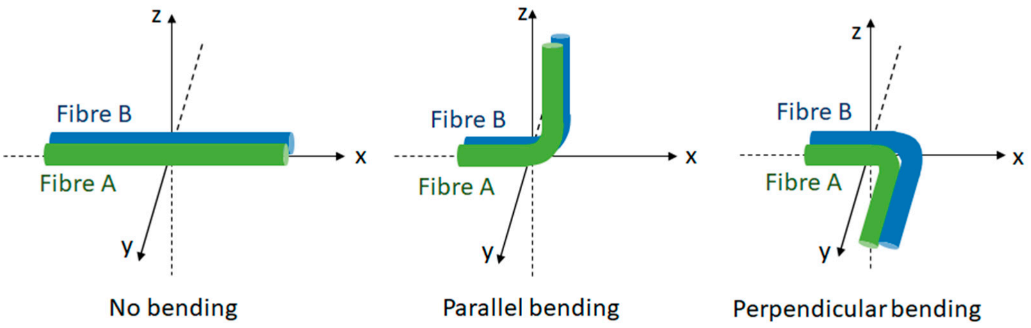

(a)

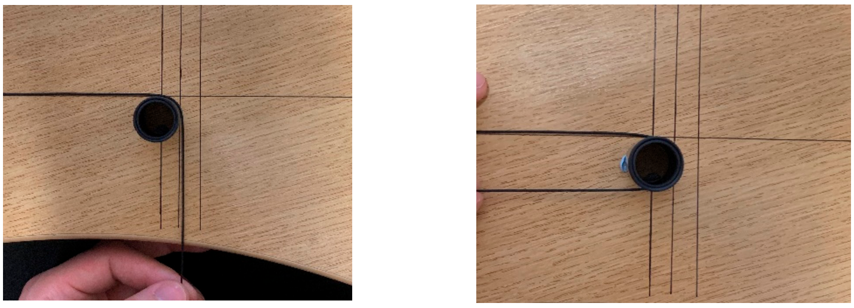

(b)

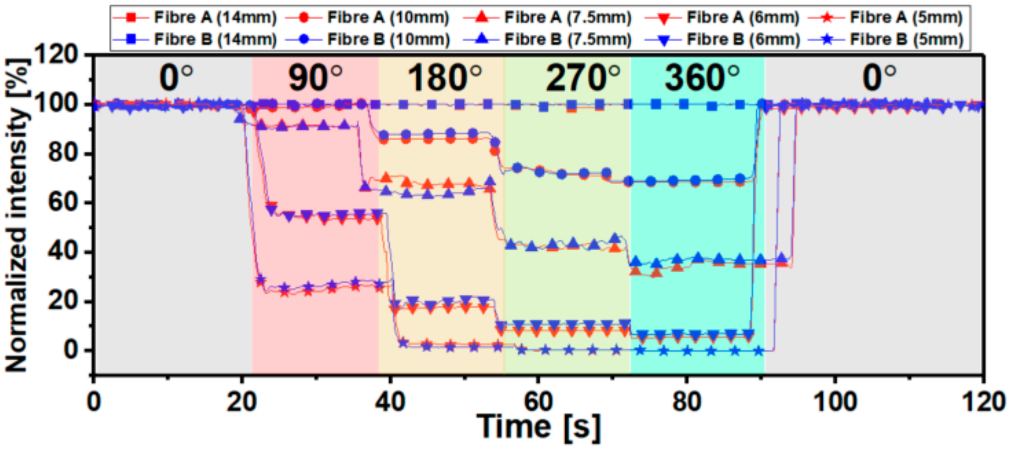

(c)

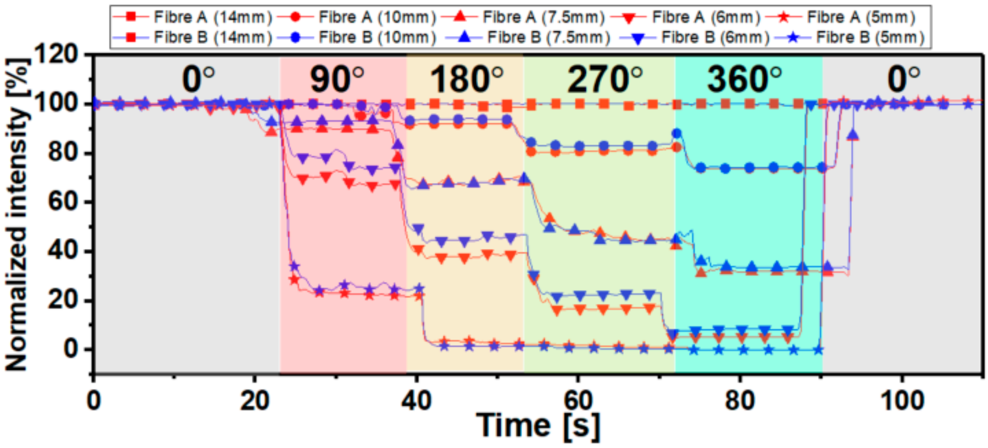

(d)

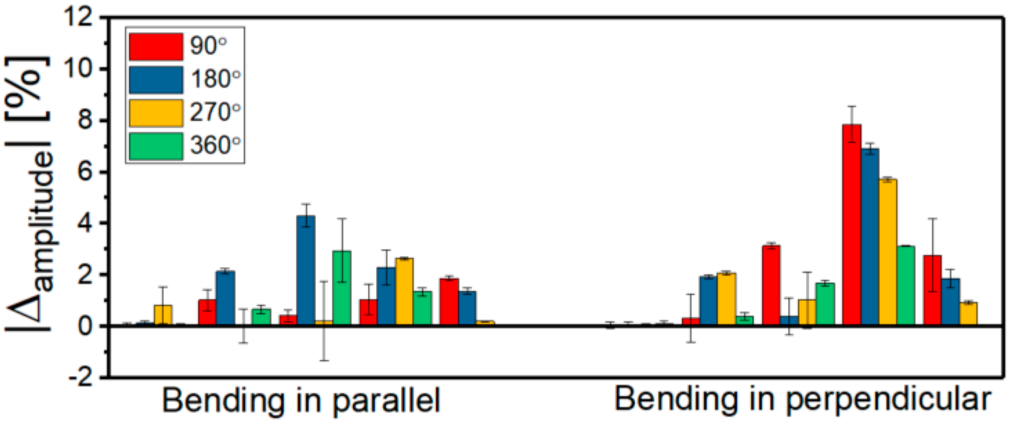

(e)

Figure S4. Bending tests with 2 bare optical fibres: (**a**) schematics of the configurations of fibres with different bending orientations; (**b**) images of the configurations of fibres with different bending orientations; (**c**) normalised intensity as a function of time in the case of bending the fibres in parallel over a range of angles around cylinders with different radii; (**d**) normalised intensity as a function of time in the case of bending the fibres perpendicularly over a range of angles around cylinders with different radii; (**e**) bar charts of differences in amplitude for bending the fibres in parallel and in perpendicular.
